# Investigating the Psychosocial Empowerment Interventions through Multimedia Education in Burn Patients

**DOI:** 10.29252/wjps.8.3.372

**Published:** 2019-09

**Authors:** Leila Mamashli, Fatemeh Mohaddes Ardebili, Tahereh Najafi Ghezeljeh, Farzad Manafi, Mehri Bozorgnejad

**Affiliations:** 1 Islamic Azad University, Ali Abad Katoul Branch, Ali Abad Katoul, Iran;; 2 Department of Medical-Surgical Nursing, Faculty of Nursing and Midwifery, Iran University of Medical Sciences, Tehran, Iran;; 3 Department of Critical Care Nursing, School of Nursing and Midwifery, Iran University of Medical Sciences, Tehran, Iran;; 4 School of Medicine, Iran university of Medical Sciences, Tehran, Iran

**Keywords:** Burn, Psychological empowerment, Multimedia, Education

## Abstract

**BACKGROUND:**

Burns patients often encounter lots of psychological problems affecting all parts of life. This study investigated the psychosocial empowerment interventions through multimedia education in burn patients.

**METHODS:**

In a randomized clinical trial study undertaken in Shahid Motahari Burn Center in Tehran, Iran in 2016, 50 patients were selected as control and 50 patients as intervention group. The demographic characteristics and the quality of life questionnaires (BSHS-B) were used. Patients in the control group received only routine educational self-care, but the intervention group received routine cares as well as the multimedia trainings. Then, the psychological quality of life was evaluated in both groups before the interventions and after three and six months.

**RESULTS:**

Before interventions, the mean of mental dimension in intervention and control groups were 2.08±0.59 and 1.64±0.47, respectively (*p*<0.001). Three and six months after the intervention, they were 3.37±0.93 and 2.24±0.4, 4.11±0.74 and 2.75±0.58, respectively (*p*<0.001).

**CONCLUSION:**

The multimedia intervention was shown to be effective in empowering the psychology of burn patients.

## INTRODUCTION

Burning is a major cause of injury and it is one of the worst destructive conditions.^1^^,^^2^ There are about 2.4 million burn cases per year worldwide that 650,000 of them need treatment, 75,000 are hospitalized and 8,000 to 12,000 are exposed to burn injuries.^3^ An epidemiological study in 2017 on 10 countries (Australia, Bulgaria, China, the Czech Republic, Finland, Iran, Israel, the Netherlands, Oman and the United Kingdom) indicated that the incidences of burnings were high in the mentioned countries.^4^ It is estimated that the incidence of severe burns in the lifetime to be around 1% and more than 300,000 deaths occur due to burns annually, worldwide.^3^

According to the National Forensic Medicine Organization, in the first quarter of 2018, 379 people died of burn, among them, 213 were male and 166 people were female, and the highest number of victims belonged to Khuzestan province with 51 deaths, then Fars province with 42 deaths, and finally Tehran city with 37 deaths.^5^ On the other hand, although having progressed in medical care the survival rate has increased in severe burns and survivors are often faced with many physical and psychological challenges.^6^

Unfortunately, the patients’ physical needs cause their psychological needs to be disregarded. The psychological trauma resulting from burnings can affect all parts of a person’s life. They cause tension in relationships, depression or drug abuse, and even more pressure on their physical health.^7^ Widespread and severe burns have a profound impact on the dimensions of life, so that 50% of the burns` are due to its latent effects.^8^ Meanwhile, the psychological disorders can last a long time,^9^ and burn itself, is a known risk factor for the development of psychiatric disorders. It may cause a number of post-traumatic factors like stress disorders, alcohol and drug use, low self-esteem, anxiety, lack of attention and support for family and friends, lack of financial support; which affects the quality of life of these patients negatively.^10^^,^^11^

Moreover, all burn victims suffer from problems such as disability, communication disorder, difficulty in wound care, decreased self-confidence, fear of their health and appearance in the future, personality change, sleep disorders, family conflicts, financial pressure and other negative feelings that may lead to social isolation while or after being discharged.^12^ It is estimated that the effects of psychological problems in patients with burns are 28-75%.^13^ These stressors can appear in a variety of ways. For example, Post Traumatic Stress Disorder Symptoms (PTSD) can be in places that are reminiscent of the incident (such as a burn center), sleep problems or having a nightmare, irritability, loss of interest in previously enjoyable activities, negative thoughts about onerself and social isolation.^7^

These problems can occur up to one year or more after being injured by burn. These facts of being emotionally devastated not only attribute to people who have experienced severe burns, but also they may include a moderate burn that does not require a lot of treatment.^7^ Not enough research has been done on the psychological consequences of burned people.^14 ^However, the survivors of burns are at a high risk of lasting mental illnesses.^15^ The need for psychological support at all stages of burn care is usually overlooked. Survivors experience a series of traumatic attacks that create great challenges for mental elasticity. They cause deep frustration and isolation.^16^ The results of a research done by Druery *et al.* (2017) indicated that social factors such as low income, unemployment, not having a house and limited social resources would affect the mental outcomes of burnt patients.^14^

Therefore, all people being burned need help and many need psychological counseling; their psychiatric care is challenging along with surgical care, maybe an intervention at the right time to be appropriate to these patients.^16^ The mental state of the patient and the expectations are effective in the comfort state after the discharge.^17^ Nowadays, great advances in care, treatment and prevention of burning have been occurred; they have focused on patient and led to the survival and improvement in the quality of burnt patients` lives.^17^ Therefore, the treatment staff should spend more energy on restoring patients to the society and home and treating the patient’s psychological consequences.^7^

In this regard, the patients should be empowered by self-care because the nurses play an important role in helping the patients with burns to adapt to new physical appearances;^3^ thus we need a nursing compiled care plan, with respect to the patient’s collaboration in self-care.^18^ Self-care is the highest priority in health and medical care systems that can provide patient satisfaction, future readiness, self-efficacy associated with illness. On the other hand, it can provide health care costs by shortening the patient’s stay in the hospital. As a result, their empowerment and adherence to treatment will increase.^19^

Nurses play an important role in improving the knowledge and empowerment of patients in clinical conditions.^20^ Accordingly, the nursing care of burn patients is of utmost importance.^21^ Nurses, as people who have close and constant contact with the patient, should find time and ability to recognize patients and choose the best way to familiarize the patients with their health and safety.^22^ But to implement the educational programs, selecting an appropriate educational method is one of the most important moves in the design process, because an effective learning is, above all, a good teaching outcome. On the other hand, one of the most important and effective factors in the quality of education is the educational methods.^23^

The problem that was presented in the current study was the inability of traditional teaching strategies and methods to achieve the educational objectives.^24^ On the other hand, the tremendous and ever-changing transformations in various educational fields and the growing need of human societies to learning and moving towards globalization have created new educational methods.^25^ In recent decades, with the emergence of new technologies, such as multimedia education the traditional approaches to learning have been undergone big changes.^26^ Furthermore, the challenges of traditional education, which was teacher-centered and the learners had a passive role, have been exposed to an essential change.^27^

The advantages of employing multimedia education include the use of multiple senses for learning, being economical, flexible against learner’s need, and so on.^28^ Multimedia education has a greater impact on the learning of individuals, because the teaching of multimedia is such that the content of the training is presented with the use of at least two media elements of the set of text, audio, image, film, animations and simulation; this will produce audiovisual stimulation and, at the same time, the individual will have access to it whenever he/she wants.^29^ Considering the issues stated and also the urgent need to pay attention to the mental dimension of burnt patients, the purpose of the current research was to determine the effect of implementing interventions based on mental empowerment through multimedia education in burn patients.

## MATERIALS AND METHODS

This research was a randomized clinical trial with a control group that was conducted in the hospitalization wards of Shahid Motahari Burn Center, Tehran, Iran in from 2016 to 2017. The study population consisted of all burn patients who were admitted to Shahid Motahari Burn Center and were participated in the study based on the inclusion criteria. The criteria for participating in the study were the patients` age, people who were between 18 to 60 years, and had the ability to use audiovisual compact discs (CDs), their burning percentage to be 10-45%, degrees of 1, 2 and 3, those having a minimum reading and writing literacy, and understanding of Persian language.

Also those who lacked sensory and motion problems and brain and mental disorders, mental retardation, those who were living in Tehran and the suburbs of Tehran, people whose burns were due to accident and non-self-immolation, non-burning with electricity were included in the current study. The exclusion criteria were the withdrawal of continued study and severity of disease, disability and death of patient. The sampling method was at convenience and continuous study; the patients were randomly assigned into intervention and control groups. 

According to the studies carried out in this regard, the effect of educational interventions with 95% of confidence and 80% of test capacity was considered on the number of samples needed for each group and taking into account 10 scores of difference in the quality of the psychological dimension of life of the two groups. So the population was estimated to be 55 people based on the following formula in a way that each group included 50 subjects, considering 10% of the probability of not participating. Finally, 100 samples were considered to be participated in the study with the formula n=2(z_1-α/2_+z_1-β_)^2^s^2^/(µ_1_-µ_2_)^2^. 

In this formula z_1-α/2_=1.96, z_1-β_=0.84, s=9 and µ1-µ2=5. All in all, two questionnaires were used in the recent study. The demographic information questionnaire and the status of the disease including some questions about gender, age, occupation, marital status, burning agent or source of heat (gasoline, gas, flame, hot liquids, oil, hot food, etc.), level of education, grade and percentage of burns, burning area, city, location of incident and economical status. This questionnaire was selected by the patient and a research associate on the first day so that the samples inclusion in the study was completed.

The next instrument was a questionnaire of burn patient’s Quality of Life [BHS-B (Burning Specific Health Scale). The psychological dimensions of this questionnaire were used. The questionnaire included 40 questions about skin sensitivity to heat, body image, hand performance, care for burnt areas, communication, ability to perform simple activities, sexual function and psychological dimension with the options of high, moderate, low and never, which had been scored from 1 to 5, respectively. Each questionnaire had at least one and maximally five scores. 

Based on this questionnaire, the quality of life in each dimension or domain was determined separately and in all domains. From 40 questions of the questionnaire, 18 questions were related to the physical dimension of quality of life, 11 questions were about the psychological dimension of quality of life and 11 questions revealed the social dimension. Demographic and disease information questionnaire was given to 10 faculty members of the faculty of medical sciences in terms of validity and content validity, and then their opinions were applied as the reliability and validity of the BHS-B were measured by Kildal *et al.* in 2001 using its dimensional analysis.^30^

In Iran, Pishnamaazi *et al.* (2009) had measured its validity and reliability by Alpha Cronbach of 94% in the burn patients in Shahid Motahari and Hazrat Fatemeh hospitals^31^ and at Qotboddin Shirazi Hospital,^11^ calculated the reliability of this tool with Alpha Cronbach of 98%. In our study, Alpha Cronbach was measured 94%. Based on the implementation of this method, the researcher referred to the Burn Medical Educational Center of Shahid Motahari Hospital after receiving the study confirmation from Iran University of Medical Sciences and the ethical code from the university’s ethics committee (93-02-28-24922-106366 on 8/12/2014 and registered in a clinical trial with the code IRCT 2014112920145.). 

After introducing the principal investigator and the collaborators of the research and the research objectives to the hospital’s officials and obtaining permission, he referred to the departments and, while introducing himself and the colleagues of the research and the study objectives to the departmental authorities, the samples were randomly provided according to the conditions of inclusion as the control or intervention group. After explaining the procedure and ensuring the anonymity of the samples, a written informed consent was obtained from each participant. They were announced that the transportation cost and the cost of left work to be compensated at 3 and 6 months by the researcher. 

Before the intervention, the demographic information and burn characteristics questionnaire was completed by the patient with the help of a research associate and using medical records. Then the intervention and control groups received face-to-face routine trainings, however, the intervention patients, in addition to routine trainings, received the self-care education of a burnt patient given at the time of discharge in an educational CD containing text, slide, film and recorded sound; then the researcher gave this CD to the patients to perform at home. In educational session they used CDs and answered questions for 30-60 minutes at the time of discharge. 

Educational content was prepared based on the sources of self-care educations of the burn patients. The questionnaire of the quality of life of burnt patients in the psychological, physical and social dimensions was completed by the patient before the intervention on the day of discharge and 3 and 6 months after the intervention; the telephone number, email address, researcher’s telegram number were given to patients to call if necessary. The researcher conducted a weekly phone contact with the patients in the intervention and control groups to follow up and ensure the preservation of the samples. 

After 3 and 6 months of intervention, the patients in both the control and intervention groups were contacted by phone to complete the questionnaire. Patients completed the questionnaires in the manner of the self-report. At the end of the research, the educational CD was provided to the control group for observing ethics in the research. After collecting raw data for the analysis, the descriptive and inferential statistics (Chi-square and independent and paired t tests for the distribution of normal variables), Fisher’s exact test, nonparametric tests such as Mann-Whitney, Wilcoxon and Friedman test and Dunn test, with Bonferroni’s correction, Spearman correlation coefficient were used by SPSS software (version 21, Chicago, IL, USA). It should be noted that all of the participants were included in the process and no one was excluded during the investigation.

## RESULTS

Among the participants of this study, 56% of the subjects were male and 56% were female. Only 34% of them in the intervention group were in the age range of 39-48 years and 44.9% of the control group`s members were in the age range of 29-38 years. According to the statistics, most of them (44% in the intervention group and 79.6% in the control group) were married. In the intervention group, 48% and in the control group, 62.5% were employed; 52.1% had diploma in the intervention group and 66.7% had diploma educational level in the control group. Moreover, 36% in the intervention group and 34% in the control group were burned by fire flame and 60% in the intervention group and 64% in the control group had a degree of burns of 1, 2 and 3.

Furthermore, 24% in the intervention group had burning percentage of 15-20% and in the control group, 36% had burning percentage of 21-26%. About 46% in the intervention group had burn in the trunk, hand, and foot and 47.9% in the control group had burn in the whole body. The majority of patients in the intervention and control group (58.1%, 79.2%, respectively) were resident in Tehran. The majority of patients in the intervention and control group (58.1% and 53.5%, respectively) were burned at home. In the intervention group, 56.5% were on average economical level and 37.8% were in weak economical level (Table 1).

**Table 1:** Demographic frequency of patients in two groups: intervention and control

**Frequency variables**		**Intervention**		**Control**		**P value**
		**No.**	**%**	**No.**	**%**	
Gender	Female	28	56.00	22	44.00	0.23
	Male	22	44.00	28	56.00	
Age	18-28	11	22.00	10	20.40	0.35
	29-38	15	30.00	22	44.90	
	39-48	17	34.00	14	28.60	
	49-58	7	14.00	6	6.10	
Marital status	Single	28	56.00	11	20.40	0.001
	Married	22	44.00	39	79.60	
Occupation	Employed	24	48	30	62.50	0.006
	Housewife	12	24	16	33.30	
	Jobless	14	28	4	4.20	
Level of education	Under diploma	2	4.20	4	8.90	
	Diploma	26	52.10	33	66.70	
	Bachelor	22	43.80	12	22.20	
	Master of higher	0	0	1	2.20	
Cause of burn injury	Gas	3	6	2	4	0.64
	Natural gas	8	16	14	28	
	Flame	18	36	17	34	
	Liquids	13	26	12	24	
	Kerosine	1	2	2	4	
	Food	4	8	1	2	
	Etc	3	6	2	4	
Percentage of burn	15-20	12	24	10	20	0.239
	21-26	12	24	18	36	
	27-32	10	20	9	18	
	33-38	8	16	2	4	
	39-45	8	16	11	22	
Degree	1,2,3	30	60	32	64	0.17
	2,3	20	40	18	36	
Area	Hands, legs	0	0	3	6.3	0.059
	Body, hands, legs	23	46	13	26	
	Head shoulder, hands, legs	10	20	11	20.8	
	Whole body	17	34	23	47.9	

The Mann-Whitney test showed that before intervention, the mean of psychological dimensions in intervention and control groups was 2.8±0.59 and 1.64±0.47, which was statistically significant (*p*<0.001). The mean difference of the score of psychological dimension in both intervention and control groups at the time before intervention was statistically significant and the mean score of psychological dimension in the intervention group was slightly higher than the control group. Mean and standard deviation of psychological dimension score in intervention and control groups three months after intervention were 3.37±0.93 and 24.2±0.4, respectively and six months after intervention, the mean and standard deviation of intervention and control group were 4.11±0.74 and 2.75±0.58, which were statistically significant (*p*<0.001) (Table 2). 

**Table 2:** Comparison of mean and standard deviation of the physical performance scores obtained for control and intervention groups before the intervention and three months after the intervention

**Group**			**Before intervention**			**Three months after intervention **		
	**Number**	**Mean**	**Standard deviation**	**Test result**	**Number**	**Mean**	**Standard deviation**	**Test result**
Intervention	50	1.61	0.71	Z=-0.82 *p*=0.41	50	3.44	0.95	Z=-6.41 *p*<0.001
Control	50	1.45	0.47		50	2.32	0.37	

**Figure 1:**
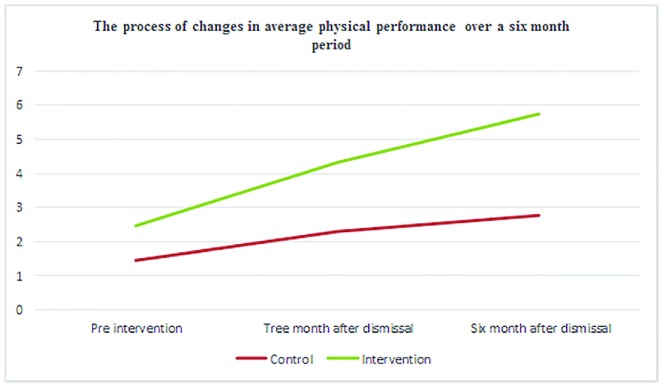
Comparison of the trend of changes in mean physical activity score before, three, and month after intervention

Considering the chi-square value (=92.50) and the value of significance level (*p*<0.001) in Table 3, since the level of significance was less than 0.05, the assumption of the equality of the mean scores of psychological dimension during three periods was rejected statistically, that is, the average score of psychological dimension varied at least in two periods of the three ones. Therefore, in order to determine which of the two periods had a significant difference, the Dunn follow-up test was used. The results of the test were presented in Table 4 and 5, while the mean score of each period had significant differences with other periods because the corrected significance level value was less than 0.05. Figure 1 shows that the psychological dimension score before intervention in the intervention group was slightly higher than the control group, but after 3 and 6 months of intervention, the psychological dimension score of the intervention group demonstrated a significant difference compared to the control group (*p*<0.001).

**Table 3:** Comparison of mean and standard deviation of the physical performance scores obtained for control and intervention groups after six months post-intervention

**Group**	**Number**	**Mean**	**Standard deviation**	**Test result**
Intervention	50	4.27	0.68	Z=-7.67
Control	50	2.76	0.56	*p*<0.001

**Table 4:** Comparison of mean and standard deviation of the physical performance scores obtained for intervention group before the intervention and at months three and six after the intervention using Friedman test

**Time**	**Number**	**Mean**	**Standard deviation**	**Test result**
Before intervention	50	1.61	0.71	Chi-Square=93.17
at month three post-intervention	50	3.44	0.95	DF=2
At month six post-intervention	50	4.27	0.68	*p*<0.001

**Table 5:** The comparison results of intervention group’s physical performance in all three times using Dunn’s pairwise test with Bonferroni’s correction

**Difference**	**Standardized test statistic**	**Corrected significance level**
Before intervention-three months after the intervention	-5.6	Adjusted *p*<0.001
Before intervention-six months after the intervention	-9.4	Adjusted *p*<0.001
Three months after the intervention- six months after the intervention	-3.8	Adjusted *p*<0.001

## DISCUSSION

The results of this study showed that interventions based on psychosocial empowerment through multimedia education in patients with burns have empowered patients in psychological dimensions. This finding was consistent with the study done by Elalem *et al.* (2018) showing that self-care nursing intervention was effective in burnt patients. Self-care intervention has led patients to participate in their treatment and have significantly improved their quality of life as well as patients’ self-confidence. Their study showed that the implementation of nursing interventions can enhance self-confidence, can improve self-image, mental health and can promote sexual relationships, all of which were considered as mental health dimensions.^3^

In another study, the researchers used educational DVDs and earned more self-confidence in exercises.^32^ Scars in burns caused the apparent deformity of the patients; consequently this deformity caused depression in the patient and problems caused by mental image and lack of self-confidence.^33^ A study conducted by Lo *et al.* (2010) showed that multimedia education (computer CDs including movies, animations and photos and brochures) reduced anxiety in patients.^34^ Psychological problems, especially anxiety in burn patients were shown to be correlated with the levels of coping, quality of life, and participation of these patients in rehabilitation activities. Anxiety was a common post-traumatic response that has been reported to a large extent in burn patients.^35^

Therefore, the patients with burns, even after receiving the most advanced remedies, often live with an abnormal appearance. Life with scar tissue, especially in social cultural conditions, which gives a great importance to the physical attractiveness, can be problematic, and the patients experience high levels of mental stress, like anxiety.^36^ Many studies suggested that one of the most common psychological problems in these patients was anxiety. Anxiety experienced in burn patients during this period resulted from the inappropriate control of pain, itching, complete body deterioration, lack of social protection systems, functional interventions and loss of autonomy.^37^

Therefore, solving psychological problems that affect the incidence of burn injury led to a further increase in the quality of life and their health.^38^ All people with burns need help and many need mental counseling.^16^ Also, early diagnosis of psychological problems, management of psychological problems in these patients and efforts to address these problems are of great importance due to the high cost imposed on individuals and society.^11^^,^^39^ Therefore, care should be considered in training programs for burn patients to maintain and strengthen patient autonomy in self-care.^40^

Meanwhile, the role of nurses in the psychological empowerment of patients is well-known.^37^ One way is to use multimedia to store large volumes of content, including images, sounds, and shapes and films; the interest in learning increases through using such media, because the eye and ear, both are involved in this skill. Instead of learning with the same speed, each person develops based on his or her own personal abilities in accordance with his or her own style and spends an appropriate time required to understand the material.^41^ Based on research findings and the impact of interventions based on multimedia education through CD on the psychological dimensions of the quality of life of burnt patients, and given that the burn patients suffer from a lot of psychological problems in this chronic and defective disease, it is necessary to help these people in the psychological self-care. 

One of these methods in empowerment could be the use of multimedia instruction, because it is economical and everywhere and can be used at any time and can easily remove the patients’ needs. Meanwhile, the family of patients can help their patients in self-care and mental empowerment according to standardized and accessible educational materials. Therefore, it is recommended that the treatment staff use this method to help these patients.
